# Practical Significance of Biomarkers in Axial Spondyloarthritis: Updates on Diagnosis, Disease Activity, and Prognosis

**DOI:** 10.3390/ijms231911561

**Published:** 2022-09-30

**Authors:** Alexandra-Diana Diaconu, Alexandr Ceasovschih, Victorița Șorodoc, Cristina Pomîrleanu, Cătălina Lionte, Laurențiu Șorodoc, Codrina Ancuța

**Affiliations:** 1 2nd Rheumatology Department, Clinical Rehabilitation Hospital, 700661 Iasi, Romania; 2 2nd Internal Medicine Department, “Sf. Spiridon” Clinical Emergency Hospital, 700111 Iasi, Romania; 3 Faculty of Medicine, University of Medicine and Pharmacy “Grigore T. Popa”, 700115 Iasi, Romania

**Keywords:** axial spondyloarthritis, diagnosis, disease activity, prognosis, systemic markers of inflammation, molecules involved in bone homeostasis, HLA-B27, newer genetic biomarkers, antibody-based biomarkers, microbiome biomarkers

## Abstract

Axial spondyloarthritis (axSpA) is a chronic inflammatory disease that can lead to ankylosis by secondary ossification of inflammatory lesions, with progressive disability and a significant impact on quality of life. It is also a risk factor for the occurrence of comorbidities, especially cardiovascular diseases (CVDs), mood disorders, osteoporosis, and malignancies. Early diagnosis and treatment are needed to prevent or decrease functional decline and to improve the patient’s prognosis. In respect of axSpA, there is an unmet need for biomarkers that can help to diagnose the disease, define disease activity and prognosis, and establish personalized treatment approaches. The aim of this review was to summarize the available information regarding the most promising biomarkers for axSpA. We classified and identified six core categories of biomarkers: (i) systemic markers of inflammation; (ii) molecules involved in bone homeostasis; (iii) HLA-B27 and newer genetic biomarkers; (iv) antibody-based biomarkers; (v) microbiome biomarkers; and (vi) miscellaneous biomarkers. Unfortunately, despite efforts to validate new biomarkers, few of them are used in clinical practice; however, we believe that these studies provide useful data that could aid in better disease management.

## 1. Introduction

### 1.1. Axial Spondyloarthritis: Past, Present, and Future

Spondyloarthritis (SpA) comprises a group of chronic immune-mediated inflammatory conditions with overlapping clinical and genetic features but with substantial heterogeneity, as inflammation can affect peripheral and axial joints and also result in extraarticular manifestations (enthesitis, dactylitis, uveitis, inflammatory bowel disease, psoriasis) [1,2,3].

SpA comprises a spectrum of diseases with significant overlaps between SpA phenotypes, which can be classified according to the Assessment of Spondyloarthritis International Society (ASAS) 2009 classification criteria. These depend on where patients primarily experience symptoms in axial SpA (axSpA) with predominantly axial manifestations, including non-radiographic (nr-axSpA) and radiographic axSpA (r-axSpA) or ankylosing spondylitis (AS) and peripheral SpA (pSpA) with predominant peripheral involvement (psoriatic arthritis, reactive arthritis, enteropathic arthritis, juvenile-onset SpA, and undifferentiated SpA) [4]. While the spectrum of axSpA extends from early or mild disease through severe or late disease with erosive damage in the sacroiliac joints, this arbitrary division of nr-axSpA and r-axSpA is becoming less clinically relevant.

The symptoms of axSpA create a significant burden for both patients and society. The major disease feature is the progression to ankylosis by secondary ossification of inflammatory lesions, with progressive disability and a significant impact on quality of life.

AxSpA is also a risk factor for the occurrence of comorbidities, especially cardiovascular diseases (CVDs), mood disorders, osteoporosis, and malignancies, which shows that axSpA has a systemic character and influences patient health in a complex manner [2]. All these characteristics affect quality of life and, because all comorbidity types correlate with axSpA, early diagnosis and treatment are needed to prevent or decrease functional decline and improve the patient’s prognosis.

### 1.2. AxSpA and Biomarkers

According to the World Health Organization, a biomarker is a chemical, physical, or biological indicator of disease presence, activity, or severity, which can provide vital information. In respect of axSpA, there is an unmet need for biomarkers that can help to diagnose the disease, define disease activity and prognosis, and establish personalized treatment approaches [5].

Despite the progress made in the concept of axSpA, its identification remains challenging, frequently resulting in a diagnostic delay for patients. Ideally, the diagnosis should be made before the development of radiographic sacroiliitis, but currently the most sensitive imaging diagnosis method is magnetic resonance imaging (MRI), which is costly and not widely available [2].

AxSpA disease activity is monitored using the following scores: Bath Ankylosing Spondylitis Disease Activity Index (BASDAI), Bath Ankylosing Spondylitis Functional Index (BASFI), and Ankylosing Spondylitis Disease Activity Score (ASDAS), which may be subjective. Disease activity is also monitored by measuring of one of the most common inflammatory biomarkers, C-reactive protein (CRP), which is insensitive for this disease [6].

Although the range of effective modern drugs for axSpA has expanded in recent decades, therapeutic decisions and the response to specific therapies remain challenges in current practice, and further research is therefore required. Adequate evaluation of disease activity, radiographic progression, response to treatment and identification of negative prognostic factors can improve the therapeutic decision. In addition, data regarding the axSpA prognosis obtained in the early disease stages will improve therapeutic management [2].

**Figure 1:**
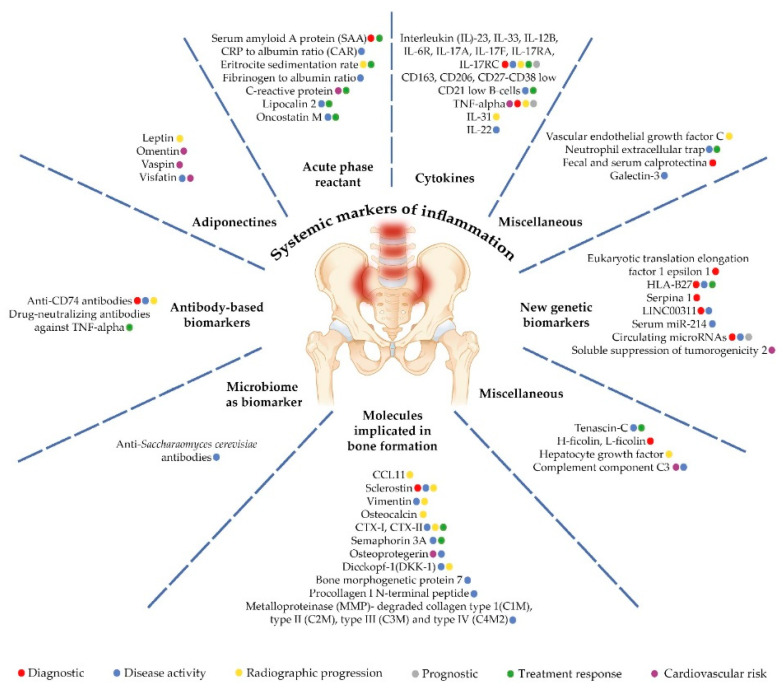
Biomarkers significance in axial spondiloarthtitis.

The aim of our review was to summarize available information regarding the most promising biomarkers for axSpA. We performed a PubMed, Embase, and Medline search for articles published in the period of 2017-2022, with “axial spondyloarthritis” and “biomarkers” as search terms. We further refer to six core categories of biomarkers: (i) systemic markers of inflammation; (ii) molecules involved in bone homeostasis; (iii) HLA-B27 and newer genetic biomarkers; (iv) antibody-based biomarkers; (v) microbiome biomarkers; and (vi) miscellaneous biomarkers (Table 1, Figure 1). Furthermore, understanding the association between biomarkers and diagnosis, disease activity, structural damage, and treatment response, we followed the same pattern when analyzing available data.

## 2. Systemic Markers of Inflammation

### 2.1. Acute-Phase Proteins

**C-reactive protein and erythrocyte sedimentation rate (ESR),** which are acute-phase proteins, are the most widely used inflammatory indicators in active disease status and have been extensively studied in respect of AS. Nevertheless, as diagnostic biomarkers, they have low sensitivity and specificity, being increased only in 40-50% of patients with AS [23]. The ASDAS-CRP score, which is used to assesses AS activity, and its subjective criteria include CRP. This score is the preferred tool for disease activity assessment in the decision-making process for axSpA treatment modification in clinical routine [73]. In addition, one of the ASAS classification criteria is high CRP values. The CRP metabolite (CRPM) is correlated with disease activity, being significantly increased in AS compared with nr-axSpA [13,71]. One recent study shows that CRP may predict spinal immobility development in AS patients [16]. Moreover, the CRP to albumin ratio (CAR), a novel inflammatory biomarker, is positively correlated with BASDAI and BASFI, being a reliable marker for assessing disease activity in patients with axSpA [23,24]. CRP levels can be predictive for axSpA treatment response [14], whereas CRP gene rs3091244 polymorphism has been found to be a reliable biomarker for a suitable response to etanercept treatment in AS [15]. Navarini L. et al. showed that persistent elevated CRP levels can be an indicator for axSpA patients at higher risk of CVD identification [13]. ESR can predict the clinical response of TNF inhibitor treatment within AS patients. In a recent study, the cut-off value of baseline ESR was 47.00 mm/h (sensitivity 57.1% and specificity 94.9%), with AUC = 0.819 [30]. In a prospective study, ESR was associated with trabecular bone score, which may be useful as a tool for assessing osteoporosis for AS [29].

**Serum amyloid A protein (SAA)** is another inflammatory biomarker that is more sensitive than other acute-phase proteins, but with lower availability and higher cost [74]. The key role of SAA in different inflammatory rheumatic conditions including axSpA is its involvement in inflammasome cascade activation and recruitment of interleukin (IL)-17-producing T helper cells [75]. SAA levels are higher in AS as compared to healthy controls and the use of a combination of SAA and CRP detection may potentially increase the likelihood of a correct and prompt diagnosis, which would enable early treatment [62]. Although there is a positive association with systemic inflammation in axSpA, and thus with disease activity, its association with treatment response is inconclusive [76].

**Pentraxin 3** is a long pentraxin overexpressed during the acute-phase response, playing significant roles in the innate immune response and inflammation, as well as in tissue damage and remodeling. Its direct implications in axSpA and many other rheumatic disorders have been extensively studied [77]; unfortunately, some studies have shown no association with disease activity and acute inflammation in axSpA [56,78].

**Fibrinogen-to-albumin ratio (FAR)** is a novel inflammatory index that is inexpensive and easily measurable, and fibrinogen is also considered an acute-phase response protein able to reflect the systemic inflammatory status [79]. Fibrinogen is an important factor involved in the coagulation system and albumin (ALB) is considered an objective indicator to foreshadow nutritional status [80]. It seems that FAR may be used as an inflammatory parameter for monitoring disease activity in AS; in a retrospective study on 135 consecutive AS patients, FAR levels were higher than in controls, with significantly higher concentrations in active disease and a positive correlation with disease activity (BASDAI score) [33].

**Lipocalin-2 (LCN-2)** is an acute-phase protein released in response to microbial triggers, which has pro- and anti-inflammatory properties [81]. In addition, **Oncostatin M** (**OSM**) is an acute-phase protein, which may be associated with lack of response to TNF inhibitors in inflammatory bowel disease (*p* < 0.00001) [82]. Both tests are involved in inflammation and bone remodeling [83]. Interestingly, persistently elevated LCN2 and OSM levels were associated with sacroiliac joint inflammation (*p* = 0.0005, respectively *p* = 0.005) in a longitudinal observational study of axSpA [51]. Moreover, clinically and serologically active patients displayed constantly raised LCN2 and/or OSM and back pain scores (> 4) after treatments [51].

### 2.2. Adipokines

Adipokines are proteins produced within white adipose tissue, with significant functions in lipid metabolism, insulin sensitivity, and energy balance regulation, and important roles in response to both infection and systemic inflammation. Due to their immunomodulatory effects, adipokines have been extensively studied in the pathophysiology of chronic inflammatory diseases, including axSpA [84].

**Leptin** is the best-known proinflammatory adipokine that can increase the direct production of several proinflammatory cytokines and may also induce the production of reactive oxygen species in macrophages, neutrophils, and endothelial cells, as well as potentiating interferon γ (IFNγ)-induced expression of nitric oxide synthetase [85]. The role of leptin in bone metabolism is complex: in the central pathway, leptin binds to its hypothalamic receptor, then inhibits osteoblast differentiation and enhances osteoclast activation by increasing sympathetic activity, while in its peripheral pathway, leptin increases bone formation and inhibits bone resorption by acting directly on osteoblasts and osteoclasts. In a longitudinal prospective study conducted in an AS population, Park J.-H. and colleagues found an increase in leptin levels that was significant from baseline to two-year follow-up and was associated with radiographic progression as reflected by the Modified Spine Assessment for Ankylosing Spondylitis Stock (mSASSS) ≥ 2. These data suggest that high serum leptin levels may reflect structural damage proportional to disease progression [48]. Another study demonstrated that biomarker combination (leptin with high molecular weight adiponectin and vascular endothelial growth factor (VEGF)) with clinical parameters (age, symptom duration, HLA-B27-positive, BASDAI, BASFI, ESR) can improve the prediction of radiographic spinal progression in SpA [49]. A recent study in AS patients reported changes in leptin and visfatin levels over the first two years and a significant association with radiographic progression after four years [66]; however, several other studies showed inconclusive results when assessing leptin’s role as a predictor of structural damage in axSpA patients [76]. Furthermore, a negative association with disease activity indices was described in respect of axSpA [76].

**Visfatin**, recognized as **nicotinamide phosphoribosyltransferase and pre-B cell colony-enhancing factor (PBEF),** is an adipokine involved in inflammation, atherosclerosis, and glucose metabolism [85]. Baykara et al. demonstrated that adipokine is significantly higher in patients with AS than in controls and correlates with BASDAI, BASFI, and carotid intima-media thickness; moreover, visfatin has been recognized to be a predictor of AS activity [72].

**Omentin-1**, known as **intellectin1,** is a novel pleiotropic adipokine involved in the inflammatory response, but is also described as an anti-atherosclerotic and cardioprotective molecule [86]; additionally, it plays an important role in bone homeostasis through the anti-osteoblastic-inhibiting and pro-osteoclastic effects of activated macrophages [87]. It seems that low omentin-1 levels are associated with metabolic dysfunction and high prevalence of cardiovascular involvement in axSpA patients [54].

**Vaspin,** an adipokine associated with CVD and systemic inflammation, was also studied in respect of axSpA. A recent study conducted on 510 patients diagnosed with axSpA suggested that vaspin is associated with cardiovascular risk and may influence the atherosclerotic process in axSpA [70].

### 2.3. Cytokines

Cytokines are small proteins predominantly produced by T helper cells (Th) and macrophages [88], and they are extensively involved in the pathobiology of different inflammatory rheumatic conditions including axSpA [89]. There are two distinct inflammatory pathways in axSpA, the tumor necrosis factor (TNF)-α axis and the interleukin (IL)-23/IL-17A axis, which have major implications for specific pathogenic events (inflammation and ossification) occurring locally (axial as well as peripheral, comprising synovitis and enthesitis) and also systemically (ocular, intestinal, cutaneous, etc.). Numerous studies have demonstrated that genetically determined high activities of the TNF-α, IL-23/IL17, and NFkB pathways are associated with increased risk of SpA [47,90]. Saif D. et al. showed that IL-17A is a valuable biomarker for detecting early axSpA changes in asymptomatic and nr-axSpA in psoriatic patients [46].

**TNF-α** is highly expressed in axSpA, especially in the sacroiliac joint biopsies of AS patients, providing a potential link between inflammation and abnormal bone homeostasis; however, different studies have shown inconclusive associations with disease activity indices and inconclusive associations with treatment responses. Thus, it seems that TNF-α is not a reliable biomarker of disease activity in axSpA [76].

**IL-6,** the second most important inflammatory cytokine in rheumatic conditions, has higher levels in AS patients than in controls, particularly in the early stages of disease, in serum, cartilage, synovial fluid, and sacroiliac joint biopsy specimens; however, studies have also shown an inconclusive association with disease activity scores, but a positive association with structural damage, as high IL-6 levels seem to predict damage in axSpA [76]. Dong, Y. et al showed that IL-6 can predict the clinical response of TNF inhibitor treatment in AS patients (the cut-off value of baseline was 9.05 pg/mL, sensitivity 76.2% and specificity 94.6%, with AUC = 0.78) [30]. Interestingly, IL-6 is associated with MRI changes; lower IL-6 serum levels relate to lower MRI inflammation scores. High baseline levels of Il-6 were highly predictive of a structural response to TNF inhibitors as assessed with the mSASSS [76].

**IL-17** has an inconclusive association with axSpA activity scores but decreases following treatment response [76].

**IL-31** serum levels correlate with sCD40L levels and mSASSS < 1, suggesting that high IL-31 is associated with reduced structural damage in early axSpA [42].

**IL-33,** a recently identified member of the IL-1 family, could be a potential candidate biomarker of disease susceptibility and anti-TNF treatment response in AS patients (*p* = 0.046, pc = NS, OR = 1.98, CI95% = 0.99–3.98) [43].

**IL-22** is a cytokine related to the IL-23/IL-17 axis and could be an independent biomarker for the differentiation of axSpA patients from non-inflammatory patients [45].

Finally, **IL-12B (rs6871626) and IL-6R (rs4129267)** gene polymorphisms could serve as promising biomarkers for diagnosis and prognosis in AS patients [44].

It is widely accepted that chronic joint inflammation in axSpA is characterized by the infiltration of activated macrophages; the **haptoglobin–hemoglobin receptor CD163** and the **mannose receptor CD206** are strongly expressed on M2c and M2a macrophages, respectively. In a prospective study, sCD206 levels were significantly higher in patients with early disease and definite radiological sacroiliitis and was significantly increased in those with a response to TNF inhibitors (*p* < 0.0001); this was not the case for sCD163 [18].

**CD27-negative B cells expressing low levels of CD21 (CD21low B cells)** are implicated in autoimmune diseases with strong B cell involvement. A recently published article emphasizes that CD27-CD38lowCD21low B cells in axSpA patients correlate positively with age and ESR. In addition, axSpA patients with and without extra-skeletal manifestations showed different frequencies of CD27-CD38lowCD21low B cells, with higher levels in those with extra-skeletal involvement [19].

### 2.4. Miscellaneous

**Calprotectin**, a DAMP (damage-associated molecular pattern) protein, is a calcium- and zinc-binding protein mainly found in the cytosol of monocytes, macrophages, and neutrophil granulocytes [91]. Recent advances suggest calprotectin as a new biomarker in inflammatory rheumatic diseases as an alternative to acute-phase proteins, since its serum levels may reflect the local activation of inflammatory innate immune cells involved in enthesitis and arthritis [92]. Serum calprotectin levels are elevated in axSpA and progressively decrease under specific medication (TNF- and IL-17A-inhibitors) [93]; in a prospective study on 451 axSpA patients, calprotectin serum levels were higher than in healthy controls and significantly associated with ASDAS as well as the presence of coxitis, suggesting calprotectin’s role in disease activity [63]. Furthermore, different papers reported raised fecal calprotectin concentrations correlating with worse disease activity (ASDAS-CRP) and visual analog scale (VAS) physical function scores [32,94]. In addition, it seems that calprotectin correlates with lipid parameters (high-density lipoprotein cholesterol, atherogenetic index) and CRP levels in axSpA patients, with subsequent implications on atherosclerosis development [65]. Calprotectin also positively correlates with CRP, ESR, BASDAI, BASFI, patient global assessment, and VAS scores, reflecting disease severity in AS patients. Moreover, those patients with elevated calprotectin levels present with higher IL-1β, IL-17, and TNF-α levels [64]. Baseline calprotectin levels correlated with mSASSS and with new syndesmophyte formation after two years according to a recent study [66]. Thus, it has been strongly demonstrated that calprotectin positively associates with disease activity indices and structural damage in axSpA, while its levels decrease following treatment response; in addition, high calprotectin predicts damage in axSpA [76].

**Neutrophil extracellular trap (NET)** is described as extracellular deoxyribonucleic acid (DNA) fibers made of histone and cytoplasmic granule proteins, involved in numerous processes, which can capture pathogens, degrade toxic bacterial factors, and kill bacteria [95]. NETosis is a mechanism of the innate immune defense playing an important role in diverse rheumatic diseases; NET-associated proteins can participate in autoimmune diseases by inducing autoantibodies against self-antigens. In a recent prospective study, neutrophils showed increased spontaneous NET formation, high expression of NET-associated signaling components, nuclear translocation of peptidilarginine deiminase 4, and elevated H3 histone citrullination in AS. In addition, patients had altered circulating levels of cell-free NETose-derived products (DNA, nucleosomes, and elastase), which positively correlated with CRP, ESR, and IL-1β concentrations. Furthermore, cell-free nucleosome levels were positively correlated with TNF-α concentration and cell-free elastase levels with CRP concentration. Interestingly, significant decreases in circulating levels of DNA, nucleosomes, and cell-free elastases were reported after six months of infliximab in a small cohort of AS patients (*p* < 0.05) [53].

**Galectin-3** is a soluble β-galactoside-binding lectin with at least one evolutionarily conserved carbohydrate-recognition domain; it performs important functions in biological activities, including cell growth, apoptosis, pre-mRNA splicing, differentiation, transformation, angiogenesis, inflammation, fibrosis, and host defense. It also seems that galectin-3 may be used as a diagnostic or prognostic biomarker for certain types of heart disease, kidney disease, and cancer [96]. In a study of 112 patients with AS and 130 healthy controls, serum galectin-3 levels were higher in AS and positively correlated with CRP, ESR, ASDAS index, and global pain index. Thus, inflammation and immune dysfunction can increase serum galectin-3 levels in AS patients, contributing to disease progression [34].

## 3. Molecules Involved in Bone Homeostasis

Understanding the pathogenesis of AS plays a key role in preventing new bone formation, one of the main causes of disability and poor quality of life. Numerous cellular elements (osteocytes, chondrocytes, immune cells, etc.), inflammatory cytokines, and cellular pathways are involved in new bone formation in AS [97]. The role of the Wnt/β-catenin pathway and its inhibitors, sclerostin and Dikkopf, has been evaluated in AS pathogenesis to identify a possible link with bone formation.

**Sclerostin** is a small glycoprotein encoded by the SOST gene primarily expressed by mature osteocytes, and is a critical regulator of bone formation [97]; it binds to the lipoprotein receptor-related protein and antagonizes the canonical Wnt signaling that normally activates TNF-α by a positive feedback loop [98]. Thus, SOST downregulates osteoprotegerin (OPG) in a dose-dependent manner, leading to an increase in the receptor activator of nuclear factor-kB ligand (RANKL)/OPG mRNA ratio, which exerts catabolic action through the promotion of osteoclast formation and activity [99]. Different studies have demonstrated lower sclerostin serum levels associated with radiographic progression in AS patients compared to healthy controls [59,100]. However, a negative association with disease activity indices was reported in axSpA [75]. The presence of circulating immunoglobulin G (IgG) antibodies that bind sclerostin (Scl-Ab) has been referred to as the cause of lower sclerostin serum levels, playing a presumed role in articular disease development. Furthermore, Scl-Ab is an emerging anti-osteoporosis treatment due to its enhanced osteogenic effects, leading to an increase in bone formation and a decrease in bone resorption [101]. In addition, high levels of Scl-Ab are associated with inflammatory bowel disease in axSpA patients [58].

**Bone morphogenetic protein-7 (BMP-7)** is an anti-inflammatory growth factor belonging to the transforming growth factor beta (TGF-β) superfamily and plays an important role in various biological processes, such as skeletal morphogenesis, embryogenesis, hematopoiesis, and neurogenesis [102]. In a prospective, multicenter French study, serum BMP-7 changes over 5 years were associated with inflammation and were elevated in active disease. In that study, serum sclerostin levels also increased significantly over time, but to a lesser extent than for serum BMP-7 [12].

**The extracellular matrix (ECM)** is a combination of collagens, glycosaminoglycans, and other molecules, but also fibers that fill intercellular spaces that undergo substantial changes during inflammation, which reflect pathological events, such as the influx of inflammatory cells. **Matrix metalloproteinases (MMPs)** can degrade ECM proteins, resulting in the release of protease-specific metabolites that can be detected as serum biomarkers of ECM tissue turnover, which would reflect local pathogenic processes. High serum MMP-3, MMP-8, and MMP-9 levels are classically reported in patients with AS and high disease activity and structural progression [103]. Type I, II, III, and IV collagens are found in the ECMs of various joint tissues (articular cartilage and hyaline, tendons, bones, and connective tissue) and can be degraded by proteases. The **MMP-mediated metabolite of type I collagen (C1M)** shows soft tissue destruction, and it is increased in AS [52]. C1M has been significantly associated with mSaSSS progression [104]. **C2M** is an **MMP-mediated metabolite of type II collagen**, which reflects cartilage destruction, while **MMP-mediated metabolite of type III collagen** (**C3M**) and **MMP-mediated metabolite of type IV collagen (C4M2)** reflect soft tissue degradation. Collagen degradation metabolite levels are higher in axSpA and these metabolites could be axSpA disease activity biomarkers. In a cross-sectional study, metabolite levels were higher in axSpA (both nr-axSpA and AS) compared to asymptomatic controls, and all metabolites except C2M were higher in AS compared to nr-axSpA. C1M, C3M, and C4M2 were associated with life quality levels, function index, and disease activity indices (ASDAS-CRP) in nr-axSpA and AS patients [52].

**Procollagen I N-terminal peptide (PINP)** is one of the most sensitive biomarkers and is particularly useful for monitoring bone formation and antiresorptive therapies. PINP is released into circulation and offers some practical benefits, including its low diurnal variability and room-temperature stability [105]. The International Federation of Clinical Chemistry and Laboratory Medicine and the International Osteoporosis Foundation recently recommended using PINP as a reference marker for bone formation in studies concerning fracture risk assessment and treatment response [106]. PINP can be useful as a marker of objective inflammation in AS, and correlates with MRI-determined SIJ inflammatory scores in AS patients but not in those with nr-axSpA [57].

**Type I and II C-terminal telopeptides (CTX-I and CTX-II)** are markers of osteoclast activity reflecting bone degradation. Different studies have shown higher serum and urinary CTX-I levels in AS patients, while CTX-II correlates with structural damage as supported by MRI inflammation scores in the sacroiliac joint and/or lumbar spine in axSpA, being considered a biomarker of radiographic progression. Remarkably, CTX-II is able to predict radiological damage and progression and correlates with changes in the mSASSS score in two-year follow-up analyses. Thus, there is a positive association of CTX-II with structural damage in axSpA, higher levels predicting further damage; CTX-II levels also predict patients at risk of developing syndesmophytes and radiographic spinal progression. In addition, CTX-II serum levels significantly correlate with disease activity and decline after TNF inhibitors, being a biomarker of treatment response in axSpA patients [76].

**Vimentin**, also known as **fibroblast intermediate filament (IF)**, is the major IF protein of mesenchymal cells, which are an important part of the cytoskeleton [107]. A specific **MMP-mediated and citrullinated fragment of vimentin** (**VICM**) could be an antigen for anticitrullinated protein antibodies that have been found in patients with r-axSpA. VICM is a measure of macrophage activity and citrullination and is higher in inflammatory diseases [71]. It seems that VICM is associated with disease activity as supported by a positive association with systemic inflammation; moreover, it is a potential biomarker of radiographic progression in r-axSpA [108], although certain studies have failed to demonstrate any relationship with mSASSS progression [71].

**Osteoprotegerin** is a member of the TNF receptor family, but it is secreted and acts like a cytokine. It is the decoy receptor for receptor activator of nuclear factor-kappaB ligand (RANKL), another cytokine that activates osteoclasts and causes bone resorption. Some studies have found elevated serum levels of osteoprotegerin [55,109] and an association with disease activity [55]. In a prospective study, osteoprotegerin levels were also associated with some metabolic syndrome features, being associated with an increased risk of CVD [55].

**Osteocalcin** is the main non-collagenous protein of bone tissue produced exclusively by osteoblasts, being a relevant marker of bone formation. Several studies have reported that high serum levels in AS are associated with radiographic progression, predicting structural damage [110,111]. However, one recent study failed to demonstrate any association between osteocalcin and ASDAS, BASDAI, BASFI and CRP levels in axSpA [112].

**Dickkopf-1 (DKK-1) protein** is a member of the Dickkopf family, composed of two cysteine-rich domains and a signal peptide sequence. DKK-1 has been acknowledged as a soluble inhibitor of the Wnt/β-catenin signaling pathway involved in bone formation through its regulation of the proliferation and differentiation of osteoblasts and osteoclasts [113]. Thus, DKK-1 participates in bone resorption or new bone formation, which are both important milestones in AS. In a recent meta-analysis, serum levels of DKK-1 were not significantly different in AS vs. healthy controls, but serum DKK-1 levels in patients with CRP > 10 mg/L and mSASSS > 30 were significantly lower than in healthy controls [114]. A prospective study showed correlation between Dkk-1 and abnormal findings in respect of MRI [25], while another study demonstrated that BMPs and Dkk-1 play an important role in the pathogenesis of abnormal bone remodeling in AS. Furthermore, Dkk-1 is higher in those taking NSAIDs regularly and bone morphogenetic proteins BMP-2/Dkk-1 significantly correlate with disease duration [26].

**Semaphorins**, which are proteins initially described as regulators of nervous system development, are involved in processes such as the regulation of immunity, angiogenesis, bone remodeling, apoptosis, and cell migration and invasion [115]. Liao H.T. et al. found increased levels of **semaphorin 3A** in AS patients compared to healthy controls and demonstrated that semaphorin 3A concentrations > 2 ng/ml are better for predicting a higher BASDAI > 4 than ESR or CRP. Furthermore, significant correlations between higher semaphorin 3A levels and the presence of uveitis, Schober’s test, and interstitial lung disease were reported in axSpA. Semaphorin 3A is also a suitable indicator for monitoring disease activity and functional status during anti-TNF treatment in axSpA (*p* = 0.013) [60]. However, Perrotta F.M. et al. found no differences between SA patients and healthy controls, but serum semaphorin 3A levels correlated positively with ESR values (*p* = 0.049) and disease activity assessed by the physician VAS (*p* < 0.01) [61].

Chemokines play an important role in immune functions and inflammatory responses, and facilitate leukocyte migration and trafficking [115].

**C-C motif chemokine ligand 11 (CCL11)**, known as an **eosinophil chemotactic protein (eotaxin-1)**, is a chemokine involved in innate immunity and inflammatory responses. CCL11 is associated with leukocyte recruitment in inflammation sites by binding to the receptor CCR3, and has been described in several chronic inflammatory diseases, brain disorders, aging, and allergic conditions [116]. In addition, inflammation-driven CCL11 expression has been shown in bone tissue as well as CCL11’s role in osteoclast migration and resorption [117]. Osteoblasts are also able to overexpress CCL11 during inflammation. Furthermore, together with its cognate receptor CCR3, CCL11 correlates with disturbed bone remodeling, and stimulates migration of osteoclast precursors and bone resorption [117]. Although the clinical significance of CCL11 in bone metabolism in AS patients has not been completely investigated, it seems that CCL11 serum levels are significantly positively correlated with mSASSS and syndesmophyte numbers [17]. Eotaxin-11 is also overexpressed in human atherosclerotic lesions [118].

## 4. Antibody-Based Biomarkers

**CD74**, an integral transmembrane molecule participating in intracellular sorting of MHC class II molecules, is involved in diverse biological processes, including the correct folding and transport of MHC class II molecules, the differentiation of B cells, and the inflammatory response as part of the macrophage migratory inhibitory factor [11]. The presence of **anti-CD74 autoantibodies** is higher in AS patients than in healthy controls [7,8,10,11]; additionally, a significant correlation between positive **anti-CD74 IgG antibodies** in patients and disease activity was demonstrated in a cross-sectional study; based on their sensitivity and specificity, anti-CD74 IgGs were suggested as promising diagnostic tools to support the clinical diagnosis of axSpA [7].

Although **anti-CD74 IgA** is elevated in patients with early axSpA, this increase is not specific enough to produce significant diagnostic value in patients under 45 years old with low back pain [10]. Anti-CD74 IgA serum levels were increased in AS and independently associated with radiological changes in the spine, suggesting that anti-CD74 IgA can play an important role in AS pathobiology [8]. Some studies show significant association with BASDAI and BASFI scores [11].

**Drug-induced neutralizing antibodies against TNF-α blockers**. Biologics are widely used to control disease, usually resulting in rapid and persistent remission; unfortunately, a significant proportion of patients do not achieve remission or fail to maintain the therapeutic response, experiencing disease flare-ups [27]. In recent years, particular attention has been paid to the formation of neutralizing antibodies during treatment with biologics, resulting in a decrease in treatment efficacy and/or safety issues. Drug-induced neutralizing antibodies against TNF-α blockers (adalimumab and etanercept) have a negative effect on the progression of inflammatory joint disease and can be used as reliable biomarkers with which to evaluate the effectiveness of treatments (*p* < 0.005) [27].

## 5. Human Leukocyte Antigen B27 (HLA-B27) and Newer Genetic Biomarkers

**HLA-B27** is an MHC class I antigen and a pivotal factor of the WHO classification criteria for axSpA. HLA-B27 testing is one of the most widely used genetic tests for any common disease, being demonstrated in up to 90-95% of AS cases compared to the general population, in which HLA-B27 is found in only 8% of cases [4]. HLA-B27 prevalence varies within different continents and ethnic/racial populations [39,40,119]. Some studies have demonstrated that HLA-B27 can predict treatment response and patients with positive HLA-B27 have a better response to TNF inhibitors than negative HLA-B27 patients [120]. HLA-B27 may also be valuable in predicting clinical manifestations, especially acute anterior uveitis; HLA-B27-positive patients have higher risk of developing anterior acute uveitis [121]. Additionally, HLA-B27-positive patients have a significantly younger age at onset, more uveitis, and a higher frequency of involvement of peripheral joints and hips than HLA-B27-negative patients [122], with a recognized progression to clinical axSpA within 1 year of follow-up as recently demonstrated [41]. However, only 2-5% of HLA-B27-positive individuals develop AS, which suggests that other genetic factors contribute to the disease [123].

**Circular RNA (circRNA)** comprises a different class of noncoding RNA produced; it is a functional molecule with important roles in regulating transcription and splicing, microRNA sponging, and modulating protein–protein interactions. CircRNA can be classified into **exonic RNAs (ecircRNAs)**, formed by exons, **circular intronic RNAs (ciRNAs)**, formed by introns, and **exon-intron circRNAs (EIciRNAs)**, formed by both exons and introns [124]. More studies have indicated that circRNAs may be associated with the risk of nerve diseases, atherosclerotic vascular diseases, cancer, and autoimmune diseases, suggesting it is a novel diagnostic and prognostic biomarker candidate, as well as a new therapeutic target for different disorders [125,126,127,128]. Recent studies reported that circRNA plays an important role in axSpA; the expression of **hsa_circ_0079787** was significantly reduced in the peripheral blood of axSpa patients as compared to healthy controls and SLE patients [129]. Moreover, Hsa_circ_0079787 was negatively correlated with BASDAI and positively correlated with the platelet count (PLT) and the lymphocyte-to-monocyte ratio. The ROC curve analysis indicated that hsa_circ_0079787 (0.715) and the combination hsa_circ_0079787-PLT-mean platelet volume (MPV)-plateletcrit (PCT) (0.835) had significant diagnostic value for axSpa. In addition, hsa_circ_0079787-HLA-B27 for axSpa vs. healthy controls was superior to that of HLA-B27 [130].

**MiR-214,** a vertebrate-specific miR precursor, is expressed in several human cells and associated with multiple diseases [131], including playing a crucial role in skeletal disorders; increased osteoclastic miR-214 is associated with both elevated serum exosomal miR-214 and reduced bone formation in elderly women with fractures [132]. In a prospective study, miR-214 levels were associated with disease activity in AS patients [67].

**Endoplasmic reticulum aminopeptidase 1 (ERAP 1)** is an aminopeptidase whose main function is to cut endoplasmic reticulum (ER) peptides to a length suitable for binding and presentation by MHC class I molecules, including HLA-B27 [133]. Various studies have demonstrated that ERAP1 is the second strongest locus associated with AS in Caucasians, just after HLA-B27 [134]. The genetic interaction between ERAP1 and HLAB27 found in AS reflects that peptide cleavage and presentation contribute to disease susceptibility [135]. A second member of the aminopeptidase family present in ER is **endoplasmic reticulum aminopeptidase 2 (ERAP2**), which significantly influences the HLA-B27:05-bound peptidome. ERAP 2 destroys some ligands and decreases the abundance of many more ligands with N-terminal basic residues, while increasing the nanometer abundance [136]. A case-control study, which evaluated the role of four single-nucleotide polymorphisms in the **ERAP1 (rs2287987, rs30187, rs27044) and ERAP2 (rs2248374) genes** and their haplotypes, demonstrated the association between these proteins and AS. The minor T allele and homozygous TT genotype of rs30187 were the most associated with AS and significantly increased disease risk, while the minor C allele of rs2287987 had a protective effect. Unlike ERAP1, no effect of rs2248374 in ERAP2 was described in relation to the disease [28].

**Long Intergenic Non-Protein-Coding RNA 311 (LINC00311)** is an RNA gene affiliated with the lncRNA class; it has been described as a critical regulator in human disease development and progression due to its role in regulating gene expression at multiple levels [137]. One study showed that lncRNA LINC00311 is involved in osteoporosis by regulating the differentiation and proliferation of osteoclasts [138]. Zhong H. and Zhong M. found that LINC00311 was upregulated in AS patients compared to healthy controls and patients with low back pain, suggesting its involvement in AS pathogenesis. They analyzed the diagnostic value of LINC00311 and showed that plasma LINC00311 may be a potential diagnostic marker for AS (AUC = 0.9041). They also reported an association with disease activity (BASDAI, ASDAS, CRP, and ESR). Interestingly, LINC00311 levels in plasma decreased after treatment, reflecting treatment outcome, and it was observed that high levels of LINC00311 showed a significantly higher rate of re-hospitalization, reflecting recurrence [50].

**Eukaryotic Translation Elongation Factor 1 Epsilon 1 (EEF1E1),** also known as **aminoacyl-tRNA synthetase-interacting multifunctional protein 3 (aiMP3/p18)**, is a protein-coding gene and is involved in AS by influencing aminoacyl-tRNA biosynthesis. Fan X. et al. suggest that EEF1E1 may be an underlying novel and important genetic biomarker for the diagnosis of AS. EEF1E1 can be controlled to promote the development of various types of cancer [139] and may also function as a tumor suppressor [140]. They also identified another crucial gene for the diagnosis of AS: serpin family a member 1 **(SERPINA1)**. SERPINA 1 is a gene that encodes alpha-1-antitrypsin (AAT) and can be correlated with AS by participating in platelet degranulation. The AAT concentration was found to be higher in patients with active-phase AS than in patients in remission/partial remission [141]. In their study, EEF1E1 and SERPINA1 levels were significantly increased and the average expression log r ratios of single-nucleotide polymorphism (SNP) sites in these genes were significantly higher in AS patients than in healthy controls [31].

**Soluble suppression of tumorigenicity 2 (sST2)** is the circulating form of the ST2L cellular receptor, expressed by cardiomyocytes and vascular endothelial cells together with its interleukin-33 ligand after cardiovascular injury [142]. It is a marker of poor prognosis in chronic inflammatory conditions [143] and a novel biomarker of cardiac fibrosis [144]. The prevalence of cardiovascular association in AS varies depending on the diagnostic method used and the pathology described, ranging from 10% to 40% [145]. Demet Ozkaramanli Gur et al. reported that sST2 can represent the link between inflammation and fibrosis in AS [35].

## 6. The Microbiome as a Biomarker

The occurrence of a joint lesion resembling reactive arthritis in a patient with bacterial dysentery prompted researchers to investigate the link between the gut and arthritis [146]. Intestinal microbial dysbiosis occurs as a common component in various inflammatory disorders, including SpA [147]. The complexity and dynamic nature of the gut microbiome in axial spondyloarthritis are under research. The gut is involved in maintaining homeostasis between the microbiota and the host, leading to the effective coordination of various immune cells [148]. Innate cells that appear to be implicated in the pathogenesis of SpA can control intestinal homeostasis by inducing apoptotic cell death and the deletion of T cells specific to activated commensal bacteria [149]. Intestinal dysbiosis is associated with worse axSpA disease activity and physical function, apparently regardless of intestinal inflammation and treatments [150]. There is no consensus regarding the bacterial species that are involved in the development of SpA. Some studies have demonstrated that microorganisms may be used as new biomarkers to characterize the pathogenesis of AS. A biomarker commonly used in the diagnosis of inflammatory bowel disease is the anti-*Saccharomyces cerevisiae* antibodies (ASCAs), yeast organisms found mainly in the small intestine. Olofsson T. et al. reported in a cross-sectional study that anti-*Saccharomyces cerevisiae* antibodies titers were elevated vs. controls, but seropositivity was not associated with disease subtype or status [32]. Another study shows distinct microbial colonization in the terminal ileum with *Lachnospiraceae*, *Ruminococcaceae*, and *Prevotellaceae* in AS patients vs. control [150]. In addition, Gill T. et al. found that *Prevotella* and *Blautia* (Lewis rats) and *Akkermansia muciniphila*, rc4-4, *Lachnospira*, and *Lachnospiraceae* (Fischer rats) are closely related to dysregulated inflammatory pathways in SpA [151].

## 7. Miscellaneous Biomarkers

The complement system is a part of the innate immune system that participates not only in the inflammatory response, coagulation, or fibrinolysis but also in the development and progression of cardiometabolic diseases [152]. axSpA is characterized by an increased risk of cardiovascular comorbidities. Therefore, the identification of biomarkers that can be used as a screening strategy to select those patients who require a closer evaluation is of considerable interest to clinicians. **Complement C3** is a potential predictor of cardiovascular events; elevated C3 levels have been closely linked to insulin resistance, abdominal obesity, and hypertension [153]. The activated form of the C3 complement can act as a hormone involved in lipid storage and energy homeostasis [154]. In a cross-sectional, Iván Arias de la Rosa et al. reported that axSpA patients with high levels of C3 complement had an increased prevalence of cardiometabolic risk factors and higher disease activity. In addition, complement C3 levels allowed the identification of insulin-resistant patients [22].

**Tenascin-C (TNC)** is a large glycoprotein of the extracellular matrix consisting of the N-terminal TNC assembly domain, 14.5 repeats similar to epidermal growth factor, repeated type III fibronectin domains, and fibrinogen-like C-terminal globular domain. TNC reflects tissue damage during the inflammatory process and also active tissue remodeling [155]. In a prospective study, it was reported that serum TNC levels are significantly higher in patients with axSpA, particularly in those with radiographic signs of advanced disease, compared to healthy subjects [68]. In addition, another study showed that TNC levels were higher in AS than in healthy controls. Gupta L et al. found that serum TNC levels had a better correlation with ESR and CRP in patients with early disease duration (≤ 5 years), and TNC decreased treatment levels in those who responded to treatment vs. those without a response [69].

**Hepatocyte growth factor (HGF)** is a multifunctional cytokine involved in embryogenesis, organogenesis, wound healing, and tissue repair and is also involved in tumorigenesis and cancer invasion [156]. HGF is expressed most by stromal cells and binds to the c-MET receptor tyrosine kinase. HGF-MET signaling can lead to a variety of cellular responses, such as morphogenesis, proliferation, cell survival, tissue regeneration, and protection against inflammatory diseases [157]. C-MET is also expressed on osteoblasts and osteoclasts; thus, HGF may regulate bone metabolism [158]. In a prospective study, HGF levels were increased in AS male patients and correlated with high mSASSS and low BMD. Moreover, HGF was positively associated with risk factors for osteoproliferation, including age, CRP, and smoking [38]. Another prospective cohort study demonstrated that elevated s-HGF levels were associated with the development of new syndesmophytes in men with AS [37].

**L- and H-ficolin** are proteins of the lectin pathway and play important roles in innate immunity participating in the clearance of apoptotic cells through complement activation [159]. In a cross-sectional cohort, H- and L-ficolin levels were increased in patients with axSpA compared to controls; in addition, the diagnostic specificity of HLA-B27 could be improved by combining it with increased concentrations of H- or L-ficolin [36].

## 8. Conclusions

Despite advances in biomarker research over recent years and continued efforts to validate new biomarkers for patients with axSpA, only a few are currently indicated in routine clinical practice, and the “classical” biomarkers still help treatment decisions via assessment of disease activity, prognosis, and response to treatment. Indeed, validation of a new biomarker usually entails a long process, but implementation requires a standardized methodology, ideal cost–benefit ratio, and definite efficiency for diagnosis, therapy, and prognosis. While higher costs and accessibility issues can limit their use, additional costs derived from a delayed diagnosis, therapeutic failure, as well as poor prognosis justify the investment.

Several newer biomarkers such as anti-CD74 antibodies, circulating microRNAs, CAR, FAR, ERAP1, EEF1E1, H-ficolin, L-ficolin, LINC00311, pentraxin 3, serum calprotectin, and tenascin-C have already expressed relevant associations with diagnosis and disease activity across multiple studies in axial spondyloarthritis. Additionally, CRP, ESR, CD163, CD206, drug-neutralizing antibodies against TNF-α, lipocalin 2, oncostatin M, NET, semaphorin 3A, and serum calprotectin properly assess response to different therapeutic agents and, thus, increase the biological therapies quality. In addition, since the sizable cardiovascular risk is widely recognized in patients with axSpA, different biomarkers are suggested, especially CRP, complement component C3, omentin-1, osteoprotegerin, sclerostin, serum calprotectin, vaspin, and visfatin serum levels for the early identification and improvement in cardiovascular outcomes in such patients.

Future studies are needed to investigate the roles and limits of the most promising serum biomarkers in providing personalized care for patients with axSpA.in the era of biologic and targeted synthetic DMARDs.

## Figures and Tables

**Table 1:** Biomarkers significance in axial spondyloarthritis.

Biomarker	Predictability	Study type	Reference
**Anti-CD74 antibodies**	Positive association with diagnosis and disease activity. Positive association with radiographic progression. Positive association with diagnosis. Negative association with diagnosis. Positive association with diagnosis.	Cross-sectional study Prospective study Prospective study Prospective study Prospective study	2021, Marwa Mahmoud Abdelaziz [7] 2021, Lan Do [8] 2019, Nelly R. Ziade [9] 2018, Janneke J. de Winter [10] 2020, Chao-Jun Hu [11]
**Bone morphogenetic protein 7 (BMP-7), sclerostin, dicckopf-1 (DKK-1)**	Positive association with disease association	Prospective multicenter study	2021, Elise Descamps [12]
**C-reactive protein (CRP)**	Positive association with cardiovascular risk Positive association with treatment response Positive association with treatment response Positive association with treatment response	Retrospective analysis Prospective study Case-control study Cross-sectional study	2022, Luca Navarini [13] 2019, Xenofon Baraliakos [14] 2020, Xu Yuansheng [15] 2018, Björn Sundström [16]
**C-C motif chemokine ligand 11 (** **CCL11)**	Positive association with radiographic progression	Cross-sectional study	2018, Dong Hyun Sohn [17]
**CD163, CD206**	Positive association with disease activity, treatment response	Prospective study	2018, Line Dam Heftdal [18]
**CD27-CD38^low^CD21^low^ B cells**	Positive association with disease activity	Cross-sectional study	2021, Rick Wilbrink [19]
**circRNA hsa_circ_0079787**	Positive association with diagnosis, disease activity	Cross-sectional study	2020, Qing Luo [20]
**Circulating microRNAs**	Positive association with disease activity Positive association with diagnosis and disease activity	Prospective study Cross-sectional study	2020, Qing Luo [20] 2018, Carlos Perez-Sanchez [21]
**Complement component C3**	Positive association with disease activity and cardiovascular risk	Cross-sectional study	2020, Ivan Arias de la Rosa [22]
**CRP to albumin ratio (CAR)**	Positive association with disease activity Positive association with disease activity	Retrospective study Cross-sectional study	2021, Zheng Zhong [23] 2021, Melih Pamukcu [24]
**Dickkopf homologue-1 (Dkk-1)**	Positive association with radiographic progression	Prospective study	2019, Zhonghai Zhao [25]
**Bone morphogenetic proteins (BMPs) and Dkk-1**	Positive association with disease activity	Prospective study	2018, Hsien-Tzung Liao [26]
**Drug-neutralizing Antibodies against TNF-*α***	Positive association with treatment response	Cross-sectional study	2020, Krasimir Kraev [27]
**ERAP1 (rs2287987, rs30187, rs27044), ERAP2 (rs2248374)**	Positive association with diagnosis	Case-control study	2019, Andrzej Wiśniewski [28]
**Erythrocyte sedimentation rate (ESR)** **ESR + Interleukin (IL)-6**	Positive association with radiographic progression Positive association with treatment response	Retrospective study Prospective study	2018, Kwi Young Kang [29] 2019, Yidian Dong [30]
**Eukaryotic translation elongation factor 1 epsilon 1 (*EEF1E1*)**	Positive association with diagnosis	Prospective study	2019, Xutao Fan [31]
**Fecal calprotectina and anti-*Saccharaomyces cerevisiae antibodies* (ASCA)**	Positive association with disease activity	Cross-sectional study	2019, Tor Olofsson [32]
**Fibrinogen-to-albumin ratio (FAR)**	Positive association with disease activity	Retrospective study	2020, Meng Liu [33]
**Galectin-3**	Positive association with disease activity	Prospective study	2019, Ming-Yu Cao [34]
**Galectin-3, soluble suppression-of-tumorogenity-2 (sST2) (cardiac fibrosis), hsCRP**	Positive association with cardiovascular risk	Prospective study	2018, Demet Ozkaramanli Gur [35]
**H-ficolin, L-ficolin**	Positive association with diagnosis	Cross-sectional cohort	2020, Anne Troldborg [36]
**Hepatocyte growth factor** **(s-HGF)**	Positive association with radiographic progression Positive association with radiographic progression	Prospective cohort study Prospective study	2021, Anna Deminger [37] 2019, Liset Torres [38]
**HLA B-27 antigen**	Positive association with diagnosis Positive association with diagnosis Positive association with diagnosis	Prospective study Prospective study Prospective study	2019, Nelly Ziade [39] 2018, Chong Seng Edwin Lim [40] 2021, Henriëtte M Y de Jong [41]
**Interleukin (IL)-31**	Positive association with radiographic progression	Longitudinal prospective cohort study	2018, Nicolas Rosine [42]
**IL-33**	Positive association with disease activity and treatment response	Prospective study	2021, Milena Iwaszko [43]
**IL-12B and IL-6R**	Positive association with diagnosis and prognosis	Prospective cohort study	2018, Wen-Feng Ruan [44]
**IL-22**	Positive association with diagnosis	Retrospective study	2022, Michal Sagiv [45]
**IL-17** **IL-23 and IL-17**	Positive association with diagnosis Positive association with diagnosis	Cross-sectional study Case-control study	2020, Dalia S. Saif [46] 2018, Jacob Sode [47]
**Leptin**	Positive association with radiographic progression Positive association with radiographic progression	Longitudinal prospective study Prospective study	2017, Ji-Heg Park [48] 2019, Judith Rademacher [49]
**Leptin + high molecular weight adiponectin + VEGF**	Positive association with radiographic progression	Cross-sectional study	2019, Judith Rademacher [49]
**LINC00311**	Positive association with diagnosis and disease activity	Prospective study	2019, Hongfa Zhong [50]
**Lipocalin 2 (LCN 2), Oncostatin M (OSM)**	Positive association with disease activity and treatment response	Longitudinal observational study	2021, Florence W.L Tsui [51]
**Metalloproteinase (MMP)-degraded collagen type I (C1M), type II (C2M), type III (C3M), and type IV (C4M2)**	Positive association with disease activity	Prospective cohort study	2019, Markéta Husšáková [52]
**Neutrophil extracellular trap (NET)**	Positive association with disease activity and treatment response	Cross-sectional study, case report	2020, Patricia Ruiz-Limon [53]
**Omentin-1**	Positive association with cardiovascular risk	Prospective study	2020, Fernanda Genre [54]
**Osteoprotegerin (OPG) and sclerostin (SCL)**	Positive association with cardiovascular risk	Prospective study	2018, Fernanda Genre [55]
**Pentraxin 3**	Positive association with disease activity	Cross-sectional study	2018, Renato Nisihara [56]
**Procollagen I N-terminal peptide**	Positive association with disease activity	Cross-sectional study	2022, Xuegang Li [57]
**Sclerostin and antisclerostin antibody**	Positive association with gastrointestinal risk Positive association with diagnosis Positive association with radiographic progression	Prospective study Case-control study Prospective study	2018, Michele Maria Luchetti [58] 2018, Perrota Fabio Massimo [59] 2019, Judith Rademacher [49]
**Semaphorin 3A (Sema 3A)**	Positive association with disease activity Positive association with treatment response	Prospective study Prospective study	2019, Hsien-Tzung Liao [60] 2017, Fabio Massimo Perrotta [61]
**Serum amyloid A1 (SAA1)**	Positive association with diagnosis	Prospective study	2020, Shijia Liu [62]
**Serum calprotectin**	Positive association with disease activity Positive association with treatment response Positive association with cardiovascular risk Positive association with radiographic progression	Prospective study Prospective study Prospective study Prospective study	2020, Matthias Jarlborg [63] 2019, Hua Hu [64] 2018, Fernanda Genre [65] 2022, Judith Rademacher [66]
**Serum miR-214**	Positive association with disease activity	Prospective study	2019, Hyun Yi Kook [67]
**Tenascin-C (TNC)**	Positive association with disease activity Positive association with disease activity	Prospective study Prospective study	2020, Kristyna Bubova [68] 2018, Latika Gupta [69]
**Vaspin**	Positive association with cardiovascular risk	Prospective study	2021, Javier Rueda-Gotor [70]
**Vimentin**	Positive association with radiographic progression	Prospective study	2019, Anne Sofie Siebuhr [71]
**Visfatin**	Positive association with cardiovascular risk Positive association with disease activity	Prospective study Prospective study	2021, Rabia Aydogan Baykara [72] 2022, Judith Rademacher [66]

## Data Availability

Not applicable.
